# Enhancing Healing in Oral Surgical Procedures: A Review on Biomaterials, Growth Factors, and Tissue Engineering

**DOI:** 10.7759/cureus.111409

**Published:** 2026-06-24

**Authors:** Avinash Sonune, Omkar Eswara Babu Danda, Sanjay Byakodi, Sonu Akhani, Nishant Visvas Dumont, Sumaiya Fatima

**Affiliations:** 1 Department of Dentistry, Sant Sevalal Maharaj Government Medical College, Washim, IND; 2 Department of Dentistry, Dr. N.T.R University of Health Sciences, Vijayawada, IND; 3 Department of Oral and Maxillofacial Surgery, Bharati Vidyapeeth (Deemed to be University) Dental College and Hospital, Sangli, IND; 4 Department of Pediatrics, Dr. N.D. Desai Faculty of Medical Science and Research, Dharmsinh Desai University, Nadiad, IND; 5 Department of Oral and Maxillofacial Surgery, Pondicherry University, Pondicherry, IND; 6 Department of Dentistry, Kamineni Institute of Medical Sciences, Sreepuram, IND

**Keywords:** biomaterials, growth factors, oral surgery, tissue engineering, wound healing

## Abstract

Oral surgical procedures require predictable healing of mucosal, periodontal, peri-implant, and maxillofacial tissues, yet repair is often challenged by microbial exposure, inflammation, mechanical stress, systemic disease, and complex defect architecture. Although biomaterials, growth factors, platelet concentrates, stem-cell therapies, hydrogels, and three-dimensional tissue-engineered constructs have been widely investigated, their clinical translation remains inconsistent because of protocol heterogeneity, variable outcome measures, limited long-term evidence, and procedure-specific differences in healing demands. This review evaluates current regenerative strategies used to enhance healing after oral surgical procedures, with emphasis on biological mechanisms, clinical applications, comparative effectiveness, translational barriers, and future therapeutic potential. Literature published between 2018 and 2026 was identified through PubMed, Scopus, Web of Science, and Google Scholar using terms related to oral wound healing, biomaterials, growth factors, platelet concentrates, stem cells, guided bone regeneration, periodontal regeneration, implant healing, and maxillofacial reconstruction. The evidence indicates that biomaterials improve wound stability, space maintenance, clot retention, and controlled delivery, while growth factors and platelet concentrates support angiogenesis, epithelial repair, fibroblast activity, matrix deposition, and bone remodeling. Tissue-engineering approaches further expand regenerative capacity through scaffold-cell-signal systems, dental stem cells, extracellular vesicles, and three-dimensional bioprinting. Overall, integrated regenerative strategies may improve oral surgical healing, but standardized protocols, robust clinical trials, and patient-centered outcomes are needed.

## Introduction and background

Healing after oral surgical procedures is a major determinant of postoperative recovery, functional restoration, and patient comfort. Delayed or impaired healing can increase pain, infection risk, functional limitations, treatment costs, and the need for additional interventions [1]. Procedures such as tooth extraction, periodontal surgery, implant placement, maxillofacial trauma repair, and reconstructive surgery require coordinated repair of mucosal, periodontal, peri-implant, and osseous tissues [2]. This clinical need is important because untreated dental disease, periodontal infection, and high oral disease burden can increase microbial and inflammatory challenges at surgical sites; high oral/dental disease burden has been associated with postoperative infective complications [1]. Delayed management of high-velocity maxillofacial injuries may worsen postoperative outcomes [3]. Time-to-operation delay has also been associated with in-hospital complications in operative facial trauma, supporting the importance of timely surgical management and perioperative optimization [4].

Systemic and local risk factors can further compromise oral surgical healing. Diabetes is especially important because it is associated with impaired host defense, periodontal disease, oral infection, and delayed wound repair [5]. Complex head and neck surgical patients may also experience wound breakdown, fistula formation, infection, and tissue necrosis, particularly after salvage surgery in tissues with reduced vascularity and compromised tissue quality [6]. These risks indicate that oral wound healing should be considered an active therapeutic outcome rather than a passive postoperative event [2].

Oral wounds differ from skin wounds because they heal in a moist, mechanically active, and microbially colonized environment exposed to saliva, food debris, biofilms, speech, and mastication [7]. Despite these challenges, oral mucosa often heals faster and with less scarring than skin, suggesting that oral tissues have distinct molecular and cellular repair pathways [8]. Comparative studies show that oral mucosal healing is characterized by rapid epithelial-cell migration, controlled inflammation, efficient extracellular matrix remodeling, and reduced fibrotic scarring [9]. The oral mucosal response to injury reflects a regulated interaction among epithelial, stromal, immune, and vascular components [10].

Normal oral wound healing occurs through overlapping phases of hemostasis, inflammation, proliferation, and remodeling. Hemostasis begins with clot formation, which limits bleeding and provides a provisional matrix for cell migration. The inflammatory phase recruits immune cells that clear debris and regulate microbial contamination. During proliferation, epithelial cells, fibroblasts, endothelial cells, and osteogenic cells contribute to epithelial closure, angiogenesis, extracellular matrix deposition, and early tissue formation. Remodeling then reorganizes extracellular matrix components and, in hard-tissue defects, supports mineralization and bone turnover [10]. Saliva contributes to healing by moistening the wound surface, buffering the local environment, providing antimicrobial components, and supporting epithelial-cell migration [7]. Dynamic changes in the oral microbiome during wound healing may influence inflammation, immune signaling, tissue remodeling, and postoperative infection risk [11]. Poor oral hygiene, inadequate nutrition, impaired host immunity, and bacterial biofilms can increase oxidative stress and inflammation, creating a wound environment that delays repair [12].

Mechanical stress is another important challenge because oral surgical sites are repeatedly exposed to speech, mastication, functional loading, and suture-line tension [2]. Regeneration of oral hard tissues is especially complex because clot stability, vascular supply, osteogenic signaling, and integration with periodontal or alveolar structures are required [13]. Conventional measures such as debridement, suturing, antimicrobial management, and postoperative education remain essential, but they may be insufficient for large defects or medically compromised patients [2]. Regenerative strategies have, therefore, been developed to actively regulate the wound environment and improve the predictability of tissue repair [13]. Biomaterials can provide space maintenance, wound protection, mechanical stability, clot retention, cell-migration support, and controlled delivery of bioactive molecules [13]. Natural and synthetic scaffolds, membranes, hydrogels, ceramics, and composite systems are increasingly used to support oral mucosal repair, periodontal regeneration, and alveolar bone repair [14].

Growth factors can support oral wound repair by regulating angiogenesis, fibroblast activity, matrix deposition, epithelial repair, and bone remodeling [13]. Platelet concentrates provide autologous fibrin matrices rich in regenerative mediators, while recombinant bioactive molecules allow more targeted biological stimulation. Tissue engineering combines scaffolds, cells, and molecular cues to improve regenerative potential in oral and maxillofacial defects [14]. Stem-cell-based and cell-free systems, including extracellular vesicles, are also emerging as promising approaches for regulating inflammation, angiogenesis, and tissue regeneration [14].

Clinical translation of regenerative oral surgical therapies remains inconsistent despite these advances. Infection control, vascularization, immune compatibility, scaffold degradation, mechanical stability, manufacturing reproducibility, cost, and regulatory complexity remain important barriers [14]. The literature is also limited by heterogeneity in biomaterial composition, platelet concentrate preparation, recombinant growth factor delivery, stem cell protocols, outcome measures, surgical indications, and follow-up duration [14]. Periodontal and alveolar bone regeneration are particularly challenging because successful repair requires restoration of an organized interface among epithelium, connective tissue, cementum, periodontal ligament, and bone [13]. This review evaluates how biomaterials, growth factors, and tissue-engineering strategies may enhance healing after oral surgical procedures, with emphasis on biological mechanisms, clinical applications, translational barriers, and future directions for biologically informed and patient-specific oral surgical care [14].

This review aims to evaluate the role of biomaterials, growth factors, and tissue-engineering strategies in enhancing healing after oral surgical procedures. It examines their biological mechanisms, clinical applications, regenerative protocols, limitations, and future potential for improving soft-tissue repair, bone regeneration, and patient-centered surgical outcomes.

Methodology

This article was designed as a narrative review to provide a clinically oriented synthesis of biomaterials, growth factors, platelet concentrates, stem-cell-based approaches, hydrogels, and tissue-engineering strategies used to support healing after oral surgical procedures. The literature was identified through targeted electronic searches of PubMed, Scopus, Web of Science, and Google Scholar. Articles published between 2018 and 2026 were prioritized to emphasize recent developments, although older foundational sources were considered when they provided essential biological or conceptual context.

Search terms included oral surgery, oral wound healing, biomaterials, growth factors, platelet-rich fibrin, platelet-rich plasma, tissue engineering, stem cells, guided bone regeneration, periodontal regeneration, implant healing, and maxillofacial reconstruction. Peer-reviewed narrative reviews, systematic reviews, randomized clinical trials, observational studies, and relevant preclinical studies were considered when they addressed oral surgical wound healing, biomaterial-based regeneration, platelet concentrates, recombinant growth factors, stem-cell therapy, tissue-engineered scaffolds, periodontal regeneration, guided bone regeneration, implant-site healing, oral mucosal repair, or maxillofacial regeneration. Articles were excluded when they were not available in English, lacked full-text access, were duplicate publications, or addressed general wound healing without clear relevance to oral, dental, periodontal, implant, or maxillofacial surgery.

Because this was a narrative review rather than a systematic review or meta-analysis, no PRISMA flow diagram, database-wise record count, quantitative synthesis, or formal risk-of-bias assessment was performed. A formal meta-analysis was not performed because the reviewed literature was heterogeneous in study design, intervention type, comparator group, surgical indication, outcome definition, and follow-up duration. Accordingly, the methods provide transparency regarding the literature search approach and thematic organization, but they are not intended to permit full reproducibility in the manner expected of a systematic review. The selected literature was synthesised thematically to cover oral wound biology, biomaterials, growth factors, tissue-engineering strategies, procedure-specific applications, comparative evidence, translational challenges, and future clinical directions. The eligibility considerations used to guide article inclusion for this narrative review are summarized in Table 1.

**Table 1:** Eligibility Considerations Used for Literature Inclusion in the Narrative Review

Screening domain	Inclusion considerations	Exclusion considerations
Language	English-language articles were considered for narrative synthesis.	Non-English articles were not included.
Publication type	Peer-reviewed narrative reviews, systematic reviews, randomized clinical trials, observational studies, and relevant preclinical studies were considered.	Editorials, letters, conference abstracts, duplicate records, and articles without accessible full text were not included.
Publication period	Articles published from 2018 to 2026 were prioritized to emphasize recent evidence.	Articles outside this period were generally not prioritized unless they provided essential biological or conceptual context.
Clinical or experimental focus	Articles addressing oral surgical wound healing, biomaterial-based regeneration, platelet concentrates, recombinant growth factors, stem-cell therapy, tissue-engineered scaffolds, periodontal regeneration, guided bone regeneration, implant-site healing, oral mucosal repair, or maxillofacial regeneration were considered.	Articles dealing only with general wound healing without clear relevance to oral, dental, periodontal, implant, or maxillofacial surgery were not included.
Narrative relevance	Articles were selected when they provided clinically relevant mechanistic, translational, or procedure-specific information for oral surgical healing.	Articles with unclear relevance to oral surgical healing or insufficient regenerative outcome information were not included.

## Review

Biology of healing in oral surgical sites

Healing of oral surgical sites begins with hemostasis, clot formation, and early inflammatory signaling. The fibrin clot limits bleeding and provides a provisional matrix that supports migration of immune cells, epithelial cells, fibroblasts, and vascular elements into the wound bed [2]. Platelets and inflammatory cells release cytokines and growth factors that initiate repair, while neutrophils and macrophages contribute to debris clearance, microbial control, and transition from inflammation toward tissue formation [10]. The oral mucosa differs biologically from skin because it has molecular and transcriptional features associated with rapid epithelial repair, controlled inflammation, and reduced scar formation [15]. This helps explain why oral mucosal wounds often heal faster and with less scarring than cutaneous wounds, despite continuous exposure to saliva, mastication, food debris, and oral microorganisms [8].

Inflammation is necessary for repair, but prolonged or excessive inflammation may delay epithelial closure, impair extracellular matrix organization, and contribute to postoperative morbidity [10]. The oral microbiome also changes during wound healing and may influence inflammatory signaling, immune response, tissue remodeling, and postoperative infection risk [11]. Bacterial biofilms can increase oxidative stress and inflammatory mediator release in the oral cavity, creating a wound environment that may inhibit tissue repair [12]. Clinical evidence further indicates that higher oral/dental disease burden is associated with postoperative infective complications, supporting the importance of oral health optimization before surgery [1].

During the proliferative phase, fibroblasts migrate into the provisional matrix and synthesize collagen, fibronectin, proteoglycans, and other extracellular matrix components that provide wound strength and structural organization [10]. Angiogenesis occurs concurrently and supplies oxygen, nutrients, immune cells, and regenerative signals to the healing site [10]. Growth factors such as platelet-derived growth factor, vascular endothelial growth factor, transforming growth factor-beta, fibroblast growth factor, and epidermal growth factor regulate fibroblast activity, endothelial-cell migration, collagen synthesis, epithelial repair, and wound remodeling [16]. These pathways are central to regenerative strategies because biomaterials, scaffolds, membranes, and delivery systems are designed to preserve space, guide cell migration, and regulate bioactive signaling [13].

Hard-tissue healing requires coordinated vascularization, osteogenesis, matrix mineralization, and remodeling. Bone repair depends on clot stability, osteoblast differentiation, extracellular matrix deposition, and remodeling by osteoblasts and osteoclasts [13]. Periodontal regeneration is more complex than simple bone fill because true regeneration requires reformation of cementum, periodontal ligament, alveolar bone, and functional connective-tissue attachment [17]. Wound-closure techniques also influence healing, and suture material can affect tissue response, microbial colonization, inflammation, and clinical wound quality [18].

Several local and systemic factors can impair oral surgical healing, including infection, delayed surgical management, diabetes, poor vascularity, nutritional compromise, smoking, medication-related effects, and extensive tissue trauma [2]. Delayed maxillofacial intervention may worsen recovery after facial trauma, while diabetes is associated with oral infection, periodontal disease, immune dysfunction, and delayed repair [3,5]. Poor tissue perfusion and biologically compromised local tissues can increase the risk of dehiscence and wound breakdown in complex head and neck surgical wounds [6]. Craniofacial wound repair occurs along a spectrum from regenerative healing to fibrotic scarring; therefore, control of inflammation, matrix remodeling, microbial burden, and regenerative signaling is important for predictable oral surgical outcomes [19].

Biomaterials for oral surgical healing

Biomaterials support oral surgical healing by stabilizing the wound, protecting the surgical site, supporting clot retention, guiding cell migration, and providing a temporary matrix for tissue repair [20]. Important material properties include biocompatibility, degradation profile, mechanical stability, handling characteristics, antimicrobial potential, and compatibility with saliva, oral microbiota, and functional loading [20]. Local hemostatic biomaterials are especially relevant after extractions, periodontal surgery, implant placement, and soft-tissue procedures because rapid bleeding control and clot stabilization are early requirements for oral wound repair [21].

Natural biomaterials are widely investigated because many resemble extracellular matrix components and can support cell adhesion, proliferation, hydration, and soft-tissue integration [22]. Collagen-based materials can act as provisional matrices and are used in plugs, sponges, membranes, and soft-tissue matrices to support hemostasis and cell migration [20]. Chitosan is of interest because of its bioadhesive, hemostatic, biodegradable, and antimicrobial properties [21]. Hyaluronic acid contributes to hydration, cell migration, inflammatory modulation, and extracellular matrix organization, making it relevant to oral mucosal and periodontal wound-healing applications [22].

Synthetic polymers, including polylactic acid, poly(lactic-co-glycolic acid), polycaprolactone, and polyethylene glycol-based systems, allow greater control over degradation behavior, mechanical strength, porosity, and drug-release properties than many natural materials [23]. These polymers are used in scaffolds, nanofiber membranes, injectable systems, and controlled-delivery vehicles for growth factors, antimicrobials, and osteogenic molecules [23]. Ceramic and mineral-based biomaterials, including hydroxyapatite, beta-tricalcium phosphate, and bioactive glass, are primarily used for hard-tissue regeneration because they can provide osteoconductive support [23]. Their brittleness and limited soft-tissue adaptability can be addressed by combining them with polymers or hydrogels to form composite scaffolds [23].

Hybrid and composite biomaterials are increasingly used because they can combine structural integrity, bioactivity, controlled degradation, and biological signaling [20]. Guided tissue regeneration and guided bone regeneration require barrier materials that limit rapid epithelial downgrowth while maintaining space for periodontal ligament cells, osteogenic cells, and vascular ingrowth [13,17]. Nanofibers, films, injectable hydrogels, and stimulus-responsive materials can improve wound conformity, sustained release, and adaptation to irregular oral defects [23]. These systems may be useful for palatal wounds and mucosal defects where moisture retention, epithelial coverage, local delivery, and protection from mechanical irritation are required [24]. Antimicrobial and bioactive biomaterial platforms are also clinically relevant because oral wounds heal in a microbially dense environment where infection and biofilm formation can delay repair [21]. Current evidence supports biomaterials as useful adjuncts in oral surgical healing, but further comparative clinical trials, standardized material characterization, and procedure-specific outcome measures are needed before broad clinical recommendations can be made [20]. Table 2 summarizes the major biomaterial categories used in oral surgical healing.

**Table 2:** Key Biomaterials Used in Oral Surgical Healing PLA: polylactic acid; PLGA: poly(lactic-co-glycolic acid); PCL: polycaprolactone; PEG: polyethylene glycol; β-TCP: beta-tricalcium phosphate

Biomaterial type	Examples	Main role	Application	References
Natural biomaterials	Collagen, chitosan, hyaluronic acid	Clot support, cell adhesion, hydration, and soft-tissue repair	Socket healing, mucosal wounds, local hemostasis, and soft-tissue augmentation	[20-22]
Synthetic polymers	PLA, PLGA, PCL, PEG	Controlled degradation, scaffold support, and controlled delivery	Guided tissue regeneration, guided bone regeneration, nanofiber membranes, and drug delivery	[23]
Ceramic materials	Hydroxyapatite, β-TCP, bioactive glass	Osteoconduction and mineralized tissue support	Ridge preservation, sinus augmentation, and alveolar bone defects	[23]
Hydrogels	Injectable and bioactive hydrogels	Moisture retention, defect adaptation, and local bioactive delivery	Palatal wounds and mucosal defects	[24]
Composite scaffolds	Polymer–ceramic or collagen–graft systems	Combined mechanical support, bioactivity, and controlled degradation	Periodontal, alveolar, and maxillofacial regeneration	[20,23]

Growth factors and bioactive molecules

Growth factors are central regulators of oral surgical healing because they influence inflammation, angiogenesis, fibroblast activity, epithelial migration, extracellular matrix deposition, and bone remodeling [25]. In oral and regenerative surgery, growth-factor-based approaches have been investigated for soft-tissue repair, periodontal regeneration, alveolar bone regeneration, implant-related healing, and other regenerative procedures [25]. Platelet-derived growth factor supports chemotaxis, fibroblast proliferation, collagen synthesis, and osteogenic cell activity, making it relevant to soft-tissue repair and periodontal regeneration [25]. Bone morphogenetic proteins promote osteoblast differentiation and mineralized tissue formation and have, therefore, been studied for alveolar and craniofacial bone regeneration [25]. Vascular endothelial growth factor contributes primarily to angiogenesis and vascular ingrowth, while transforming growth factor beta regulates inflammatory responses, fibroblast proliferation, extracellular matrix deposition, and wound remodeling [25,26].

Platelet concentrates are clinically relevant because they provide autologous growth-factor-rich preparations within fibrin-based matrices [25]. These preparations include platelet-rich plasma, platelet-rich fibrin, advanced platelet-rich fibrin, leukocyte-platelet-rich fibrin, and concentrated growth factor, each of which may support clot stability, soft-tissue healing, postoperative recovery, and early regenerative signaling [25,27]. Randomized clinical evidence has shown that concentrated growth factor constructs can improve wound healing after third molar surgery [27]. A systematic review also reported that concentrated growth factors may reduce postoperative sequelae and enhance healing outcomes after third molar extraction, although effects vary according to preparation method, surgical indication, and study design [28]. These findings suggest that platelet concentrates may be most useful when the clinical priority is soft-tissue recovery and reduction of postoperative symptoms after selected oral surgical procedures [27,28].

Recombinant growth factors offer a more targeted approach by delivering selected bioactive molecules at controlled concentrations [25]. Recombinant platelet-derived growth factor and bone morphogenetic proteins have been investigated for periodontal regeneration, alveolar bone development, and repair of osseous defects [25]. Their clinical performance depends on appropriate case selection, carrier design, local retention, and biologically suitable dosing [25,26]. Growth factors may also be incorporated into bioactive coatings on implants and scaffold surfaces to support localized regenerative signaling [26].

Controlled-release systems are important because growth factors may have limited biological half-life and may diffuse or degrade rapidly at oral surgical sites [26]. Hydrogels, membranes, microspheres, nanoparticles, collagen matrices, and coated implant or scaffold surfaces can be used to retain growth factors locally and regulate their release [26]. These delivery systems may improve local bioavailability while reducing the need for high or repeated doses [26].

Clinical limitations remain important. Growth-factor therapies may be limited by dose variability, cost, lack of protocol standardization, rapid diffusion, uncertain long-term safety, and inconsistent clinical responses [25]. Insufficient control of delivery may also increase the risk of undesirable tissue responses, including ectopic mineralization or excessive fibrosis [26]. Future research should focus on standardizing platelet-concentrate protocols, optimizing biomaterial carrier systems, conducting rigorous randomized clinical trials, and developing procedure-specific delivery strategies based on oral wound biology [28]. Table 3 summarizes major growth factors, platelet-derived products, and bioactive adjuncts used to support oral surgical repair.

**Table 3:** Growth Factors and Bioactive Molecules in Oral Surgery PDGF: platelet-derived growth factor; BMPs: bone morphogenetic proteins; VEGF: vascular endothelial growth factor; PRP: platelet-rich plasma; PRF: platelet-rich fibrin; CGF: concentrated growth factor

Molecule/system	Main function	Delivery method	Application	References
PDGF	Fibroblast activity, chemotaxis, collagen synthesis, and osteogenic support	Recombinant form or platelet-derived preparations	Periodontal and soft-tissue repair	[25]
BMPs	Osteoblast differentiation and mineralized tissue formation	Grafts, scaffolds, or carrier-based delivery systems	Alveolar and craniofacial bone regeneration	[25]
VEGF	Angiogenesis and vascular ingrowth	Bioactive coatings or controlled-release carrier systems	Vascular support during mucosal and bone healing	[26]
PRP/PRF/CGF	Autologous growth-factor release and fibrin-matrix support	Platelet-concentrate matrix	Third molar surgery, socket healing, and selected regenerative procedures	[25,27,28]
Controlled-release growth-factor systems	Local retention and sustained bioactive signaling	Hydrogels, membranes, microspheres, nanoparticles, collagen matrices, or coated surfaces	Growth-factor delivery in oral regenerative procedures	[26]

Tissue engineering strategies

Tissue engineering in oral surgery is based on the scaffold-cell-signal concept, in which biomaterials provide structural support, cells provide regenerative capacity, and biochemical cues regulate proliferation, differentiation, angiogenesis, and matrix formation [29]. This approach has been investigated for periodontal defects, alveolar bone loss, oral mucosal defects, peri-implant defects, and maxillofacial reconstruction, particularly where conventional grafting may be limited by donor-site morbidity, material availability, or incomplete tissue integration [30]. Scaffolds are designed to maintain space, support cell attachment, guide vascular ingrowth, and degrade at a rate compatible with new tissue formation [31]. Hydrogels are especially relevant in oral tissue engineering because they are injectable, hydrated, flexible, adaptable to irregular defects, and capable of delivering cells, growth factors, antimicrobials, or extracellular vesicles within the wound microenvironment [31].

Mesenchymal stem cells are widely studied because they can support osteogenic, chondrogenic, fibroblastic, and cementoblastic differentiation and may also release immunomodulatory and pro-angiogenic signals [29]. Dental and craniofacial stem-cell populations are of particular interest because they may provide site-relevant regenerative potential for oral and maxillofacial tissues [30]. Periodontal ligament stem cells are relevant to periodontal regeneration because they may contribute to cementum, periodontal ligament, and alveolar bone formation [29]. Dental pulp cells and gingival-derived cells have also been investigated for dentin-pulp repair, mucosal regeneration, and soft-tissue engineering applications [30].

Three-dimensional bioprinting has expanded tissue-engineering possibilities in maxillofacial reconstruction by enabling patient-specific scaffold architecture, spatial cell distribution, and defect-matched constructs [32]. Bioprinted constructs can be designed with controlled porosity, mechanical gradients, and localized bioactive-molecule distribution to support bone formation and integration in anatomically complex defects [32]. Patient-specific design is particularly relevant in craniofacial surgery because defects are often irregular and require precise anatomical reconstruction for functional and aesthetic restoration [32].

Cell-free regenerative approaches are emerging as alternatives to direct cell transplantation. Stem-cell-derived extracellular vesicles and exosomes can deliver proteins, lipids, and nucleic acids that regulate inflammation, angiogenesis, osteogenesis, and matrix remodeling [29]. These strategies may reduce challenges related to cell survival, immune response, and manufacturing complexity while preserving paracrine regenerative effects [29].

Major barriers remain. Engineered oral tissues require adequate vascularization to prevent necrosis, especially in large bone or mucosal defects [30]. Functional regeneration may also require innervation for sensation, pain regulation, and integration with surrounding oral tissues [14]. Routine clinical application will require improved scaffold mechanics, antimicrobial protection, reproducible cell processing, controlled degradation, and stronger clinical validation [31]. Table 4 summarizes current tissue-engineering and procedure-specific regenerative strategies for oral and maxillofacial healing.

**Table 4:** Tissue Engineering and Procedure-Specific Regenerative Strategies MSCs: mesenchymal stem cells; PDL: periodontal ligament; PRF: platelet-rich fibrin; 3D: three-dimensional

Strategy	Main components	Target use	Key outcome	References
Scaffold-cell-signal engineering	Biomaterials, cells, and growth cues	Periodontal, mucosal, and bone defects	Guided tissue repair and regeneration	[29,30]
Dental stem-cell approaches	Periodontal ligament, dental pulp, and gingival-derived cells	Periodontal, dentin-pulp, mucosal, and craniofacial repair	Site-relevant tissue regeneration	[29,30]
MSC-based tissue-engineering approaches	Mesenchymal stem cells with scaffold and signaling systems	Oral and maxillofacial regenerative applications	Regenerative support, immunomodulation, and tissue repair	[29,30]
3D bioprinting	Bioinks, cells, and patient-specific scaffold design	Patient-specific maxillofacial defects	Anatomical reconstruction and defect-matched tissue support	[32]
PRF-biomaterial combinations	PRF with grafts, membranes, or scaffold materials	Socket preservation, ridge augmentation, and maxillofacial regenerative procedures	Clot stability, angiogenic support, and scaffold-assisted regeneration	[33,34]

Procedure-specific applications

Regenerative oral surgery is increasingly tailored to the biological requirements of specific procedures [2]. In extraction sockets, key therapeutic objectives include clot stabilization, soft-tissue closure, preservation of alveolar ridge volume, and reduction of infection or excessive post-extraction bone resorption [33]. Platelet-rich fibrin combined with grafts, membranes, or other biomaterials may support socket healing by improving fibrin architecture, growth-factor release, angiogenic signaling, and early matrix formation [33]. These combinations are especially relevant for ridge preservation because they combine the biological activity of platelet concentrates with the space-maintaining function of grafting biomaterials [34].

Third molar surgery requires rapid soft-tissue healing, control of pain and swelling, and protection of the extraction wound from microbial contamination [2]. Platelet-rich fibrin is a three-dimensional autologous biomaterial that may support soft-tissue healing, clot stabilization, and sustained release of regenerative mediators at the operative site [34]. Topical oxygen therapy has also been proposed as an adjunctive strategy in implantology, oral surgery, and periodontology because oxygen delivery may support wound healing and help control bacterial burden in selected clinical contexts [35].

Resveratrol has been investigated as a bioactive adjunct for bone regeneration because of its reported antioxidant, anti-inflammatory, and osteogenic properties relevant to oral and maxillofacial applications [36]. Periodontal and peri-implant surgery require procedure-specific regenerative planning because clinical outcomes depend on defect morphology, soft-tissue stability, inflammatory control, biomaterial selection, and long-term tissue integration [37]. Plasma rich in growth factors has been studied as an adjunct in intraoral bone grafting procedures, where its potential value is linked to biologically active mediators that may support early bone healing [38]. Platelet-rich plasma has also been evaluated in oral surgery, but systematic-review evidence indicates that clinical effects vary because of differences in preparation protocols, platelet concentration, activation methods, surgical indications, and outcome assessment [39]. Resorbable biopolymers are another important biomaterial group in oral surgery, although their effectiveness and complication profile depend on polymer type, surgical indication, handling characteristics, and clinical context [40].

Implant-site regeneration requires adequate bone volume, stable soft-tissue coverage, microbial control, and predictable peri-implant wound repair [35,37]. Biomaterial-assisted regeneration and platelet-rich fibrin-biomaterial combinations may support peri-implant or ridge-augmentation procedures by improving clot stability, scaffold support, and local regenerative signaling [33,37]. Periodontal regenerative therapy requires restoration not only of bone but also of cementum, periodontal ligament, and functional connective-tissue attachment [13]. Platelet-rich fibrin-biomaterial combinations may provide both biological stimulation and scaffold support for periodontal wound repair [33,34].

**Figure 1:**
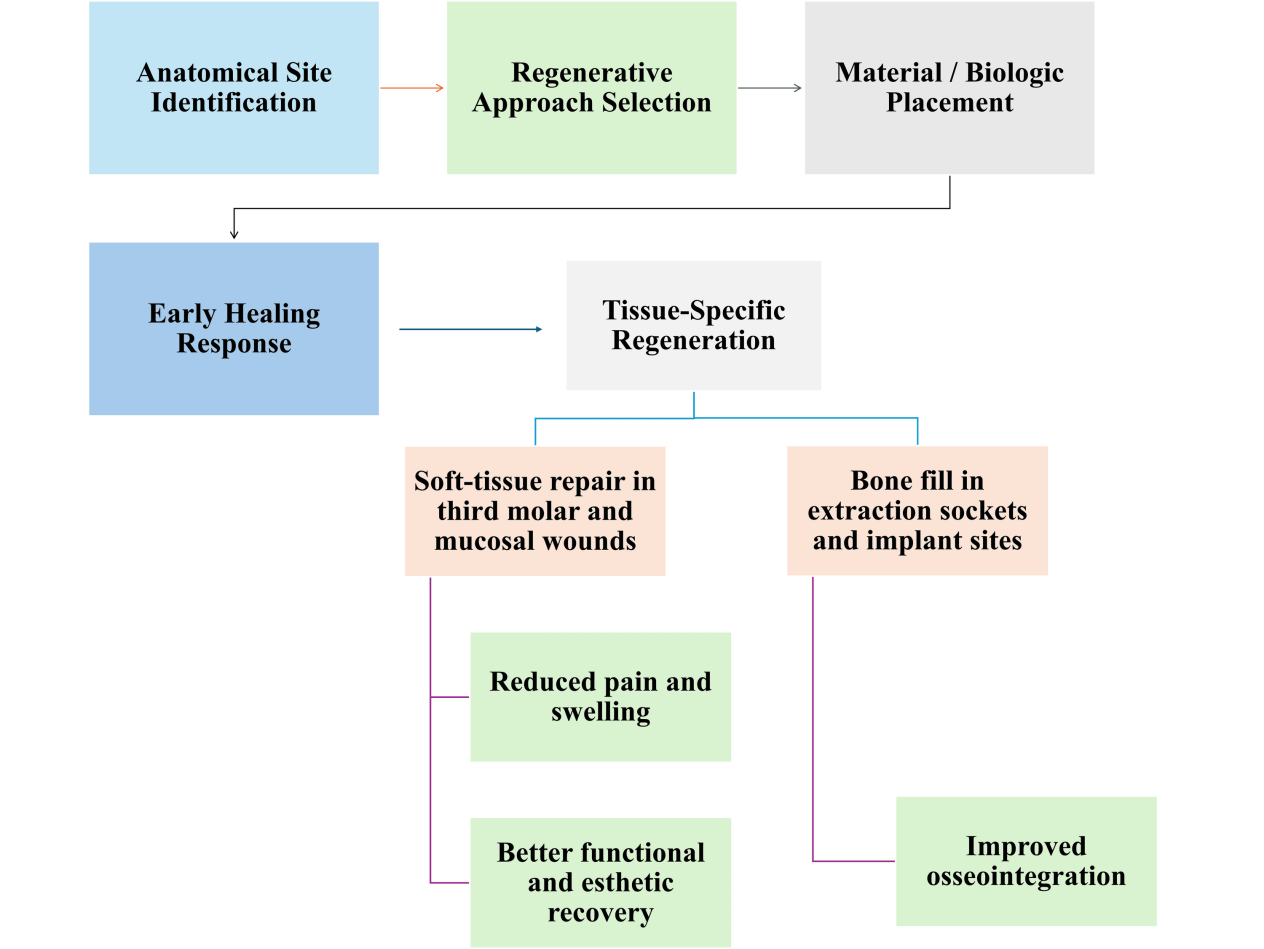
Comparative Evaluation of Current Regenerative Approaches in Oral Surgery Created by authors using Microsoft PowerPoint

Guided bone regeneration and sinus-augmentation procedures depend on maintenance of regenerative space, barrier function, vascular ingrowth, and gradual replacement of graft materials by new bone [33,38]. Large maxillofacial defects, including cleft, cystic, traumatic, and reconstructive defects, require regenerative approaches that support volume maintenance, vascularization, and integration with native bone [36]. Adjunctive strategies such as platelet-rich fibrin, topical oxygen therapy, resveratrol-based approaches, plasma rich in growth factors, platelet-rich plasma, and resorbable biopolymers may support selected aspects of oral mucosal or bone repair, although their indications and clinical effectiveness differ by procedure and evidence level [34-40]. Current regenerative strategies, as illustrated in Figure 1, vary in biological potential, clinical benefits, and limitations.

Comparative evaluation of current approaches

Current regenerative approaches in oral surgery can be broadly grouped into biomaterials, biologics, and combined systems, each with distinct clinical advantages and limitations. Biomaterials primarily support space maintenance, clot stabilization, mechanical support, and tissue organization, whereas biologics primarily modulate cellular signaling, angiogenesis, inflammation, and matrix formation [37]. Combined approaches may provide broader regenerative support by incorporating platelet-derived products or other bioactive mediators into grafts, membranes, or scaffolds, although their effectiveness remains dependent on the procedure, defect type, and clinical endpoint being assessed [37,38].

Autologous biologics such as platelet-rich plasma and plasma rich in growth factors are clinically attractive because they are patient-derived and contain multiple regenerative mediators in a usable chairside or surgical preparation [38]. Plasma rich in growth factors has been investigated in intraoral bone-grafting procedures, but the evidence remains limited by heterogeneity in protocols, defect types, outcome measures, and follow-up periods [38]. Platelet-rich plasma has also been widely evaluated in oral surgery, but systematic-review evidence indicates that its clinical efficacy is inconsistent across studies because of variation in preparation methods, platelet concentration, activation protocols, surgical indications, and outcome assessment [39]. Recombinant growth factors provide more defined biological activity and dosing than autologous platelet-derived preparations, but their use requires appropriate carriers, local retention, and dose control to reduce the risk of inconsistent responses or unwanted tissue effects [25,26].

Natural and synthetic scaffolds differ in biological behavior and clinical handling. Natural scaffolds may support cell compatibility, extracellular matrix similarity, and tissue integration, but their degradation profile, mechanical strength, and batch reproducibility may be less predictable [20,22]. Synthetic and resorbable biopolymers allow greater control over structure, degradation kinetics, and mechanical performance, although their clinical value depends on biocompatibility, inflammatory response, handling properties, and complication profile [40]. Systematic evidence suggests that resorbable biopolymers can be useful in oral surgery, but outcomes and complication rates vary according to polymer type, surgical site, and clinical indication [40].

**Figure 2:**
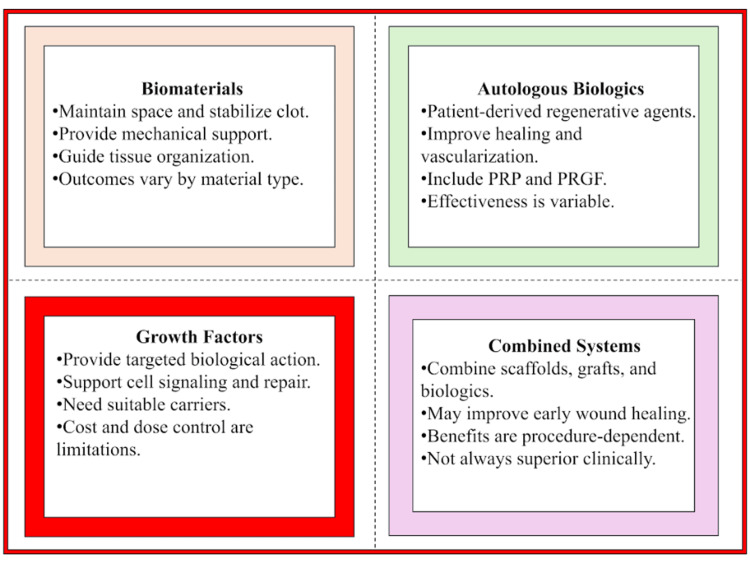
Procedure-Specific Applications of Regenerative Strategies in Oral Surgery PRP: platelet-rich plasma, PRGF: plasma rich in growth factors Created by authors using Microsoft PowerPoint

Regenerative approaches should be evaluated through procedure-specific outcomes rather than broad material categories alone. In periodontal and peri-implant surgery, relevant outcomes include true tissue regeneration, clinical attachment gain, bone fill, probing-depth reduction, soft-tissue stability, and long-term survival of teeth or implants [37]. Patient-centered outcomes, including pain, swelling, trismus, wound healing, function, esthetics, and quality of life, are also important when assessing regenerative adjuncts in oral surgery [39]. Overall, the evidence supports regenerative materials and biologics as potentially useful adjuncts, but clinical predictability remains limited by methodological heterogeneity, small sample sizes, inconsistent follow-up, and insufficient standardized comparative trials [37-40]. Regenerative strategies are, therefore, selected according to the biological requirements of each oral surgical intervention, as shown in Figure 2.

Translational and clinical challenges

Clinical, regulatory, manufacturing, and ethical barriers continue to limit the translation of stem-cell-based and regenerative approaches into routine oral and maxillofacial surgery. Mesenchymal stem cells have shown potential for bone repair, soft-tissue regeneration, periodontal reconstruction, and maxillofacial defect repair, but the clinical evidence remains heterogeneous because studies differ in cell source, culture method, delivery system, indication, and outcome measures [41]. Dental stem cells are of particular interest because their craniofacial origin may support regeneration of periodontal ligament, dentin-pulp complex, alveolar bone, and oral mucosa; however, standardized clinical application remains limited [42].

Standardization is a major challenge for biologic therapies. Stem-cell products and platelet-concentrate preparations may differ in cell source, processing method, centrifugation protocol, cell or platelet concentration, activation method, scaffold carrier, and local delivery strategy [43]. This heterogeneity makes cross-study comparison difficult and reduces reproducibility in clinical practice [43]. Cell-based regenerative products also require controlled processing, sterility assurance, product identity testing, potency assessment, traceability, and compliance with good manufacturing practice requirements before clinical use [44]. Current overviews of mesenchymal stem-cell use in facial bone regeneration further emphasize that clinical translation requires careful control of cell processing, scaffold selection, vascularization, and integration with native bone [45]. These requirements increase cost and restrict advanced regenerative therapies to specialized clinical or research settings [41,44].

Biomaterial translation also requires control of sterility, degradation kinetics, mechanical stability, and biological compatibility. Scaffolds must maintain regenerative space, avoid premature collapse, degrade at a rate compatible with tissue formation, and avoid excessive inflammatory response [42]. These requirements are particularly important in oral surgery because biomaterials are exposed to saliva, microbial biofilms, masticatory forces, and irregular defect geometries [42]. For facial bone regeneration, biomaterials and cell-based constructs must also support vascularization and integration with native bone while maintaining volume and anatomical contour [45].

Scalability and cost-effectiveness remain unresolved. Autologous cell harvesting, laboratory expansion, quality control, transport, storage, and chairside delivery can make stem-cell therapies expensive and operationally complex [44]. Ethical concerns include donor-cell sourcing, informed consent, degree of cell manipulation, long-term safety, commercialization, and premature clinical marketing before sufficient evidence is available [43]. Small cohorts, short follow-up, variable endpoints, and limited randomized controlled trial evidence further restrict clinical certainty [41]. Larger multicenter trials using standardized protocols, validated outcome measures, and long-term follow-up are needed before stem-cell and advanced regenerative therapies can become predictable components of oral surgical care [44].

Limitations and future directions

This review has several limitations. Because it was designed as a narrative review rather than a systematic review or meta-analysis, it did not include a PRISMA flow diagram, database-wise record counts, quantitative synthesis, or formal risk-of-bias assessment. This limits strict reproducibility because the number of records identified, screened, excluded, and finally included was not formally tracked. Therefore, the findings should be interpreted as a clinically oriented synthesis rather than a comprehensive systematic appraisal of all available evidence. Across the included literature, study design, biomaterial type, growth-factor preparation, stem-cell source, surgical indication, comparator group, follow-up duration, and outcome assessment were highly heterogeneous, which limited the feasibility of meta-analysis or meta-regression. Many studies were preclinical or based on small clinical cohorts, which limits direct comparison and reduces the strength of clinical recommendations. Protocol variability was especially evident in platelet-concentrate preparation, scaffold fabrication, growth-factor dosing, and stem-cell processing. Long-term safety, cost-effectiveness, degradation behavior, immune response, and patient-centered outcomes were also inconsistently reported.

Future research should prioritize well-designed multicenter randomized clinical trials using standardized protocols and validated outcome measures. Future systematic reviews may further stratify evidence by study design, intervention type, comparator group, clinical indication, and outcome quality to permit more formal quantitative synthesis where appropriate. Greater attention is needed for procedure-specific applications, including extraction sockets, periodontal defects, implant sites, mucosal wounds, and maxillofacial reconstruction. Advanced biomaterials should be optimized for antimicrobial activity, vascularization, mechanical stability, and controlled bioactive release. Stem-cell-based and cell-free therapies require stronger regulatory frameworks, reproducible manufacturing methods, and long-term monitoring. Patient-specific regenerative strategies based on individual risk factors may improve the predictability of oral surgical healing.

## Conclusions

This review suggests that biomaterials, growth factors, and tissue-engineering strategies are valuable regenerative approaches for enhancing healing following oral surgical procedures. Oral wound healing occurs within a complex environment, which is affected by saliva, microbiota, inflammation, mechanical stress, and patient-specific risk factors. Biomaterials can enhance wound stability, facilitate clot retention, create regenerative space, guide cell migration, and serve as carriers of bioactive molecules. Growth factors and platelet concentrates stimulate angiogenesis, fibroblasts, epithelial repair, extracellular matrix deposition, and bone remodeling. Regenerative possibilities are also extended by using tissue-engineering techniques that incorporate the use of scaffolds, stem cells, hydrogels, controlled-release systems, and three-dimensional bioprinting. The evidence is currently supporting the use of these strategies in extraction socket healing, periodontal regeneration, implant-site development, oral mucosal repair, guided bone regeneration, and maxillofacial reconstruction. The predictability of clinical outcomes remains limited because of heterogeneous protocols, irregular biomaterial properties, irregular outcome measures, cost, complexity of regulation, and a lack of long-term randomized clinical trials. Further research is needed to focus on standardized procedures in preparation, procedure-specific protocols, multicenter clinical validation, and patient-centered outcomes. Generally, the biological guided formation of regenerative material, bioactive mediators, and tissue-engineering ideas may help to improve the quality of healing, functional rehabilitation, and long-term outcomes in oral surgical care.
